# Pb/Pb_3_O_4_ Metal–Semiconductor Nanocomposite Obtained on 4A Zeolite—Optical and Structural Properties

**DOI:** 10.1021/acsomega.3c07247

**Published:** 2024-02-09

**Authors:** Ricardo Britto-Hurtado, Eduardo Larios-Rodriguez, Rafael Ramírez-Bon, Luis Patricio Ramírez Rodríguez, Temístocles Mendívil Reynoso, Mario Flores Acosta, M. Cortez-Valadez

**Affiliations:** † CONAHCYT-Departamento de Investigación en Física, Universidad de Sonora, Apdo. Postal 5-88, 83190 Hermosillo, Sonora, Mexico; ‡ Departamento de Ingeniería Química y Metalurgia, Universidad de Sonora, Rosales y Luis Encinas S/N, 83000 Hermosillo, Sonora, Mexico; § Centro de Investigación y de Estudios Avanzados del IPN, Unidad Querétaro, Apdo Postal1-798, 76001 Querétaro, Qro, Mexico; ∥ Departamento de Física, Universidad de Sonora, Apdo. Postal 5-88, 83190 Hermosillo, Sonora, Mexico; ⊥ Departamento de Investigación en Física, Universidad de Sonora, Apdo. Postal 5-88, 83190 Hermosillo, Sonora, Mexico

## Abstract

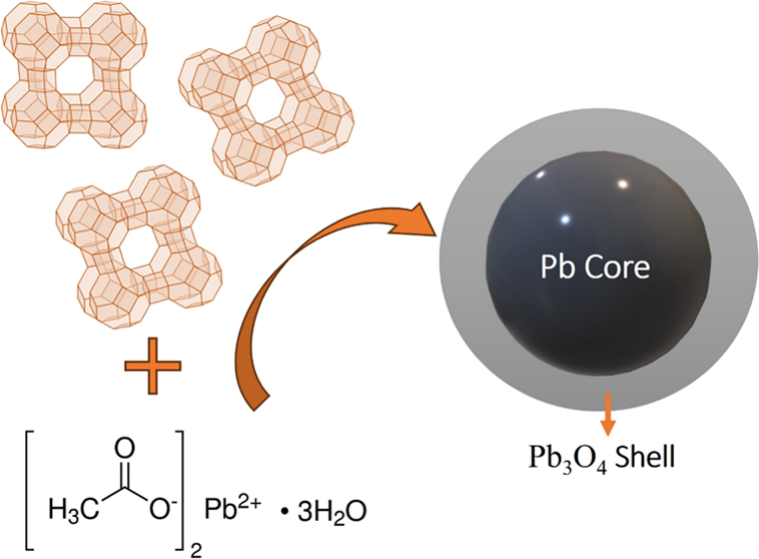

This work describes a controlled and low-cost synthesis method to obtain Pb/Pb_3_O_4_ nanocomposites using synthetic zeolite 4A. The nanostructures obtained have a core–shell configuration with 5–25 nm diameters. High-resolution transmission electron microscopy (HRTEM), BF, high-angle annular dark-field annular scanning transmission electron microscopy (HAADF-STEM), energy-dispersive X-ray spectroscopy (EDS), X-ray photoelectron spectroscopy (XPS), and ultraviolet–visible (UV–vis) characterization techniques were used. Crystallographic planes (111), (200), and (220) for the core and planes (110) and (211) for the shell, corresponding to FCC and tetragonal structures for Pb and Pb_3_O_4_, respectively, were determined using HRTEM. The HAADF-STEM images allowed the analysis of intensity contrast images proportional to the number of atoms. XPS spectral analysis showed a 4.8 eV difference in binding energy between Pb 4f_7/2_ and Pb 4f_5/2_ for lead and lead oxide. EDS elemental mapping, XPS, and UV–vis spectroscopy analyses revealed the simultaneous presence of lead and lead oxide in the same structure. The band gap obtained for the shell was determined to be 4.50 eV. Consequently, Pb/Pb_3_O_4_ nanocomposites show a higher response to high-energy photons, making them suitable for UV photocatalysis applications.

## Introduction

Metallic nanoalloys containing metal oxides can enhance mechanical properties, thermal stability, electrical and magnetic characteristics, and corrosion resistance when compared to individual nanomaterials or their larger-scale counterparts.^1,2^ These improvements enable a wide range of specific applications, especially in solar cells, biosensors, catalysis, and nanodevices, among others.^3−6^ In addition, incorporating metal oxides can provide special electrical or magnetic properties to the alloys.^7−9^ Lead and its oxides are mainly used to manufacture rechargeable lead-acid batteries, corrosion-resistant surface coatings, and catalysts.^10−12^ In this context, photoconductive lead oxides have gained popularity as essential semiconductor materials in various optoelectronic devices.^13−15^ Among the lead oxides, minimum or red lead (Pb_3_O_4_) is a semiconductor with a band gap around 2.1–2.2 eV. It stands out due to its excellent pyroelectric and ferroelectric properties and high electrical resistivity.^16^ Individual synthesis of Pb and Pb_3_O_4_ nanoparticles has been reported to have interesting applications. Recently, the synthesis of Pb nanoparticles incorporated on a carbon surface with sizes <5 nm using the well-known reductant sodium borohydride (NaBH_4_) was reported to improve advanced lead–carbon battery systems.^17^ Elango and Roopan reported using a green synthesis method to obtain lead nanoparticles with sizes of 47 nm with antimicrobial and photocatalytic activity.^18^ On the other hand, the synthesis of Pb_3_O_4_ nanoparticles with average particle sizes of 40 nm, synthesized by reaction of lead nitrate with hydroxide for catalytic applications, was reported.^19^ Also, metal or polymer alloys with lead and lead oxide have been used in various applications due to their unique properties. These alloys can exhibit a combination of characteristics of both components, making them suitable for specific uses. Taha reported Pb_3_O_4_/PVC nanocomposites prepared by the solution-casting route to improve the thermal stability of PVC films.^20^ Danish et al. recently reported the synthesis of Pb_3_O_4_/Co_3_O_4_ nanocomposite using the modified reverse microemulsion process for electrochemical applications.^21^ Ullah et al. reported the synthesis of ZrO_2_/Pb_3_O_4_ rod-shaped nanocomposites by the sol–gel method and sintering at 400 °C.^22^ Likewise, lead (Pb) and lead dioxide (PbO) systems are widely used in the catalytic processes. For example, the Pb/PbO_2_ compound is used as electrodes in electrochemical cells, such as battery cells.^23,24^ The PbO_2_ in the electrodes can improve the efficiency and stability of the redox reactions in these cells. There are very few literature reports on Pb/PbO nanocomposite systems. Khanuja et al. reported Pb/PbO core–shell nanostructures with diameter sizes of 30 nm.^25^ Hsu et al. reported superconducting Pb/PbO nanoparticles with a Pb core of 5 nm and a PbO layer of 1.2 nm.^26^ Recently, a Pb/PbO compound confined in mesoporous carbon (OMC) was used as electrocatalysts for the electroreduction of CO_2_ to CO.^27^

Furthermore, reports on synthesizing nanocomposites of porous materials or zeolites with lead or lead oxides are limited. Dapurkar SE reported preparing and characterizing metal oxide nanoparticles, including PbO nanoparticles within the mesoporous channels of silicate molecular sieves MCM-41 and MCM-48.^28^ Lead and lead sulfide nanoparticles were previously reported in zeolite X (F9) and natural zeolite (clinoptilolite), respectively.^29,30^

Zeolite 4A has several advantages for nanomaterial synthesis over natural zeolites. For example, zeolite 4A has a precise and uniform chemical composition, allowing reproducible and consistent experiments.^31^ Natural zeolites can have different compositions due to geological conditions.^32^ In addition, natural zeolites have a lower ion exchange capacity than zeolite 4A, which has a larger specific surface area and, therefore, a higher ion adsorption capacity.^33^ In this case, we report a convenient and controllable synthesis method for obtaining Pb/Pb_3_O_4_ metal–semiconductor nanocrystals using 4A zeolite, which does not require sophisticated techniques or catalysts. According to our inquiry, this would be the first report of this synthesized nanocomposite.

## Materials and Methods

### Experimental Section

Commercial synthetic zeolite 4A from Waco Chemicals Inc. with formula Na_12_[(SiO_2_)_12_ (AlO_2_)_12_]_27_·H_2_O was used to prepare the samples. This zeolite has a sodalite cavity size of 0.6 nm with a 0.4 nm window. The incorporation of Pb into the zeolite was carried out in the following steps

**Chart 1:**
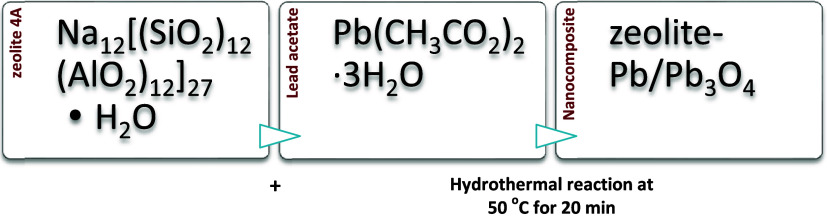
Schematic Diagram of the Three-Step Synthesis Procedure to Obtain the Pb/Pb_3_O_4_ Nanocomposite

(a) Zeolite hydration: In this stage, deionized water is added to the zeolite with a ratio of 1 g/10 mL of water, with magnetic stirring. This part is helpful to eliminate air in the zeolite cavities.
(b) Exchange process: 20 ± 0.01 mL of 0.07 M lead acetate (Pb(CH_3_CO_2_)_2_·3H_2_O, Sigma-Aldrich, 99,9% purity) are added for 20 min at 50 °C, this stage is to induce the Pb^2+^ exchange with the zeolite. Subsequently, a wash was performed with deionized water to remove unbound ions. The sample was dried under a vacuum at a low temperature. Not using heat treatment in the drying process avoided energy input to the system. Finally, the zeolite-Pb/Pb_3_O_4_ system is obtained (see Chart 1).

#### HRTEM Analysis

The nanoparticle solution was dispersed in ethanol, and a drop of this suspension was deposited on a holey carbon holey grid. The samples were characterized using the JEOL-JEM2010 transmission electron microscope operated at 200 kV. Mean Pb/Pb_3_O_4_ particle size and structural analysis were obtained using filtered image profile images by selecting specific fast Fourier transform (FFT) reflections.

#### STEM Analysis

High-angle annular dark-field annular scanning transmission electron microscopy (HAADF-STEM) is a technique with chemical sensitivity and has high spatial resolution. The samples were analyzed on a JEOL-JEMARM200F (with a resolution of 78 pm) electron microscope operating at 200 kV, with a CEOS corrector for the condenser lens. Z-contrast STEM images were recorded simultaneously in the BF and HAADF modes. Images were recorded with a condenser lens aperture of 40 μm (angle of convergence 32–36 mrad). Chemical analyses were performed with energy-dispersive X-ray spectroscopy (EDS) and EELS. EDS analysis was performed by EDAX. Spectral line scanning and chemical maps were obtained by using EDAX Genesis software. A probe size of 7C (75 pA) and a Cl aperture size of 40 μm were used for EDS analysis. The probe size used was 6C (145 pA), and the aperture size was 40 μm, with a camera length of 6 cm.

#### XPS and UV–Vis Analysis

PERKIN-ELMER Model PHI5100 X-ray Emitted Photoelectron Spectrometer, with Mg and Al source. The optical properties were measured with a Perkin Elmer Lambda 19 UV/vis/NIR spectrophotometer.

## Results and Discussion

**Figure 1:**
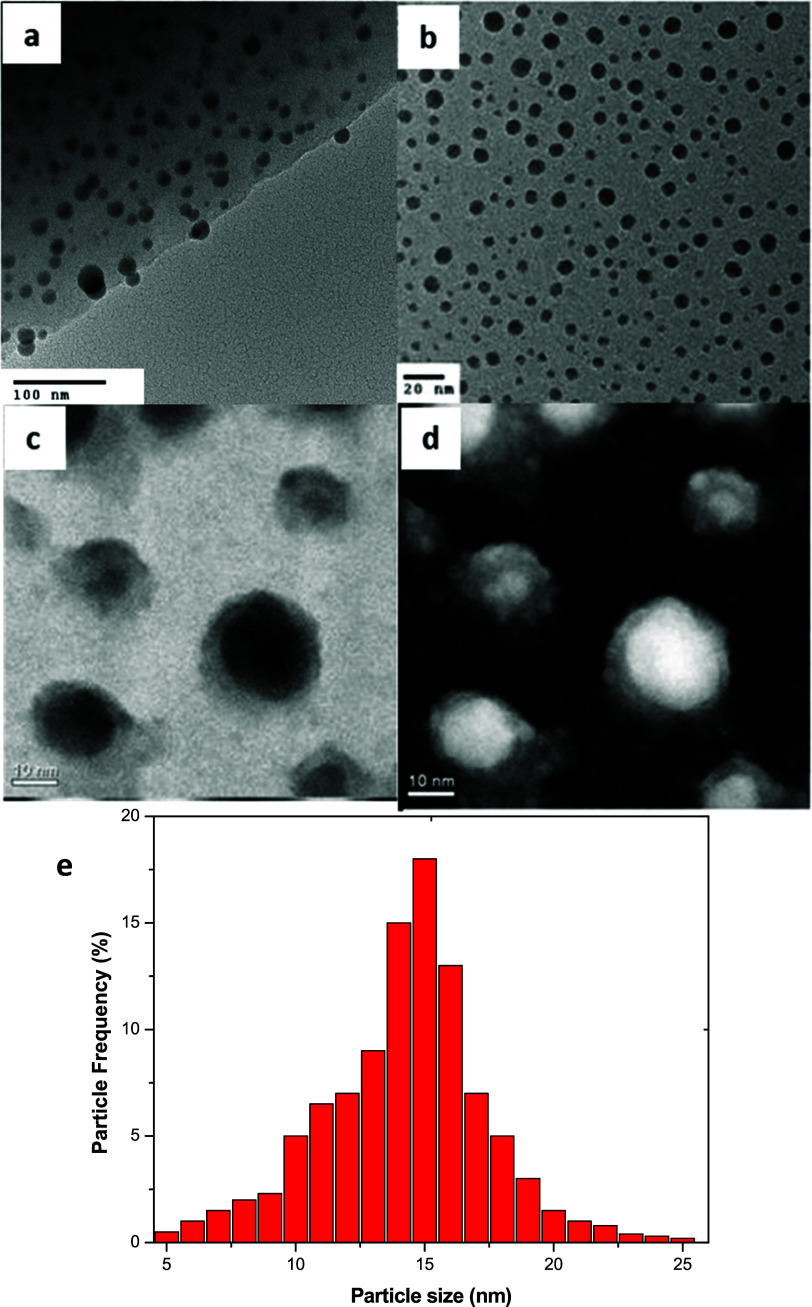
(a, b) Low-magnification TEM images of Pb@Pb_3_O_4_ nanoparticles. (c, d) Medium magnification of BF and HAADF-STEM, respectively. (e) Size distribution of Pb_3_O_4_ nanoparticles.

Figure 1a,b shows two low-magnification TEM micrographs of the synthesized nanostructures in zeolite 4A. Figure 1c,d shows BF and HAADF-STEM images of Pb/Pb_3_O_4_ at medium magnification. It is observed that the nanoparticles have a core–shell type structure, showing a remarkable contrast between the core and the shell. The differences in intensities are related to the particle sizes (different thicknesses). Pb/Pb_3_O_4_ nanoparticles with 5–25 nm diameters are observed (Figure 1e).

**Figure 2:**
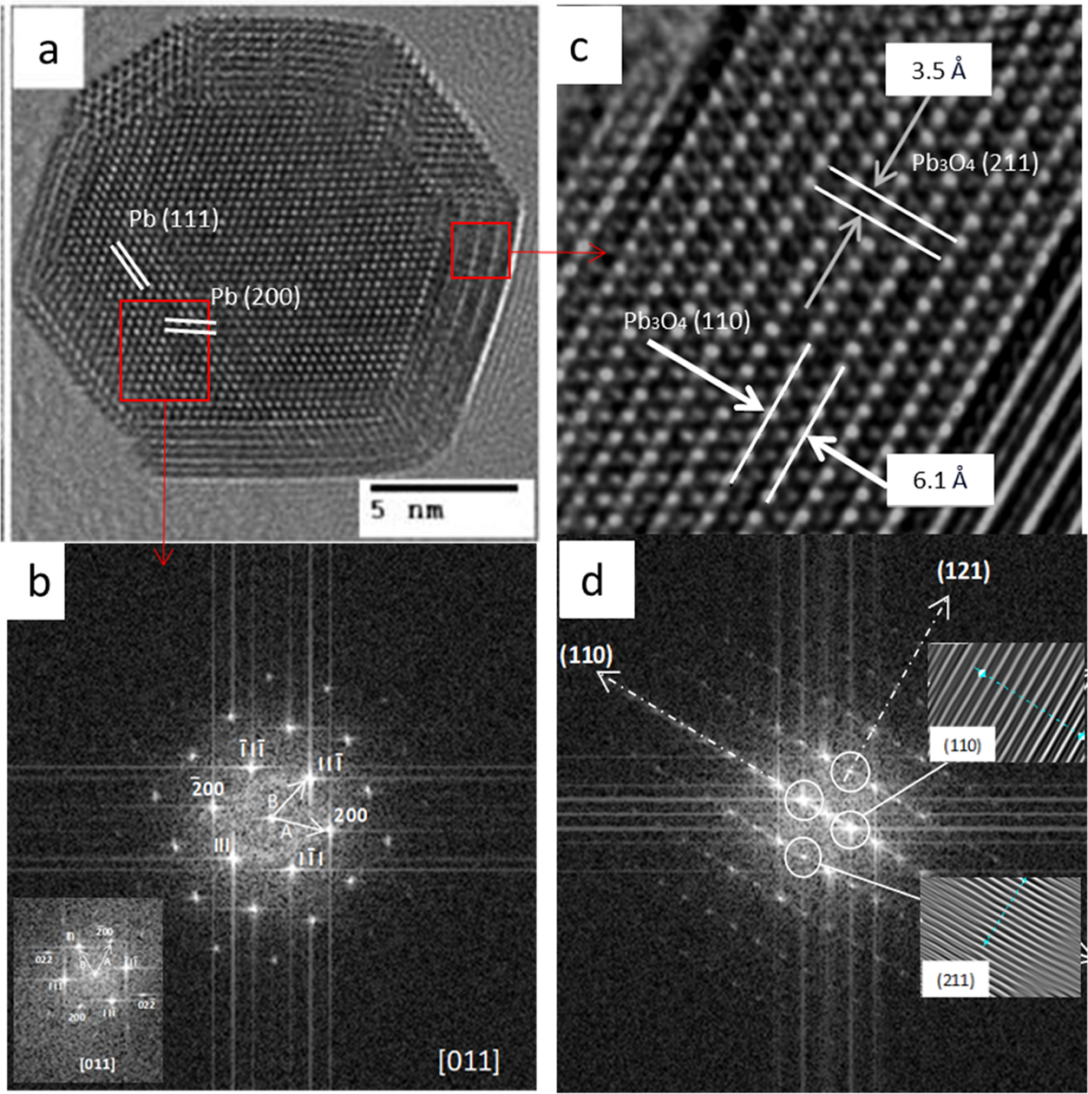
(a) High-resolution TEM image of Pb/Pb_3_O_4_ nanoparticles. (b) FFT of the nanoparticle core (square area). (c, d) FFT of nanoparticle shell and calculation of *d*-spacings using DigitalMicrograph.

Figure 2a shows an high-resolution transmission electron microscopy (HRTEM) image of the Pb/Pb_3_O_4_ nanoparticle. Image 2b shows FFTs of the area marked with a square on the nanoparticle. The reflection patterns indicate that the ratio between the distances A and B is 1.15, and the angles between A-B and B–B are close to 54.74 and 70.52°, respectively. Therefore, this means that the patterns obtained by FFT correspond to a (011) FCC crystal structure. The reflections corresponding to the (111) family of planes correspond to a 2.7 Å fringe spacing of the FCC structure. Reflections corresponding to family (200) correspond to the FCC structure’s fringe spacing of 2.39 Å.

In comparison, reflections corresponding to family (220) also correspond to the FCC structure’s fringe spacing of 1.69 Å. The microscope was previously calibrated by using a gold standard in imaging and diffraction modes to measure lattice spacing. We measured the lattice spacing in several images using DigitalMicrograph software from the FFT images of the HRTEM micrographs. After measuring different zones, we found an average of 2.71, 2.4, and 1.6 Å for the (111), (200), and (220) families of planes, respectively. These correspond to the *d*-spacing indicated in the JCPDS files for FCC Pb, File no.:01–073–7077, which present a lattice parameter of 4.8 Å. Figure 2c shows an HRTEM micrograph of the fully oxidized shell. The atomic arrangement of the crystal structure is completely different from that of the observed core in Figure 2a for the FCC structure of Pb. Figure 2c,d shows a magnified image of the marked box in the nanostructure and the pattern obtained by FFT, respectively. An interplanar spacing of 6.1 Å corresponding to (110), perpendicular to the (110) reflections, and the interplanar spacing of 3.5 Å corresponding to (121), also perpendicular to their respective reflections, were obtained. These values of 6.1 and 3.5 Å correspond to the spacings reported in the JCPDS files for tetragonal Pb_3_O_4_ (File No 01–073–6505), which present lattice parameters *a* = *b* = 8.7 Å and *c* = 6.55 Å.^34^ Pb and other oxides, such as PbO, PbO_2_, PbO_1.44_, and Pb_2_O_3_, have markedly different lattice parameters. Table 1 summarizes the values measured from the FFT analysis and the family of planes corresponding to these spacings.

**Table 1:** Interplanar Spacing and Crystal Structures of the Pb Core and Pb_3_O_4_ Shell

measures *d*-values (Å)	*hkl*/spacing (Å)	element/compound	structure
6.1	110:6.207	Pb_3_O_4_	tetragonal
3.5	211:3.37	Pb_3_O_4_	tetragonal
2.7	111:2.77	Pb	fcc
2.39	200:2.402	Pb	fcc
1.69	220:1.698	Pb	fcc

**Figure 3:**
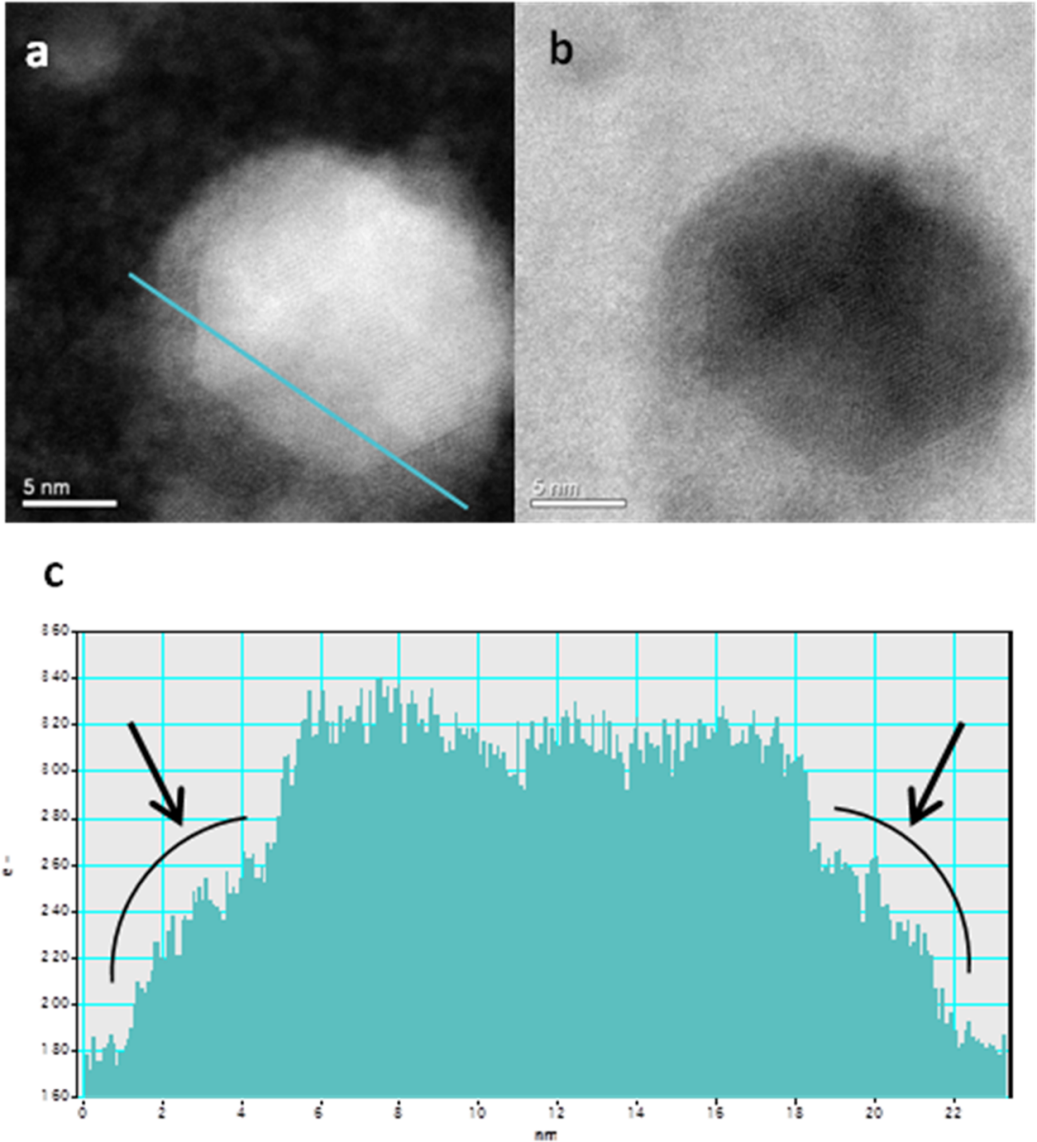
(a, b) High-magnification BF and HAADF-STEM images of Pb/Pb_3_O_4_ nanoparticle indicating core–shell composition, arrows show intensity for Pb_3_O_4_ (shell). (c) Pb/Pb_3_O_4_ nanoparticle profile.

Figure 3a,b shows a representative Pb/Pb_3_O_4_ core–shell nanoparticle in BF and HAADF-STEM images. A perfectly defined hexagonal-shaped core is observed and clearly shows discontinuity at the edges, indicating a noticeable intensity step shown by arrows (Figure 3c). Since HAADF intensity is proportional to atomic number, this significant HAADF-STEM intensity step can be explained by a different composition between the core and the shell. Therefore, the contrast difference is associated with the high-intensity core corresponding to Pb, while the lower-intensity shell corresponds to the compound Pb_3_O_4_.

**Figure 4:**
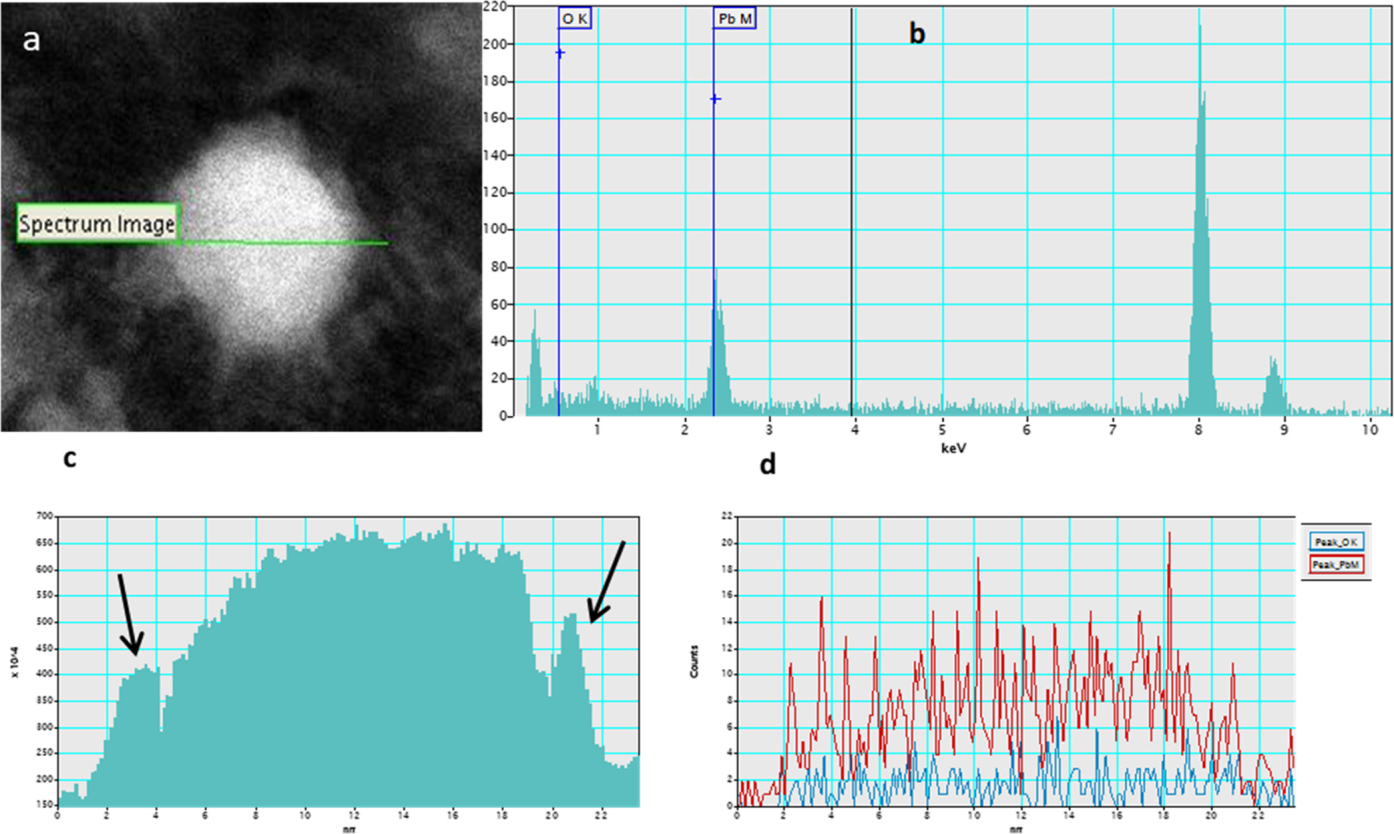
(a) HAADF-STEM image spatial drift for EDS (b) EDS line scan performed across a single Pb/Pb_3_O_4_ core–shell nanoparticle shown in (b). (c) Line scan analysis carried out on the line from (a), showing Pb-core (more intensity) and Pb_3_O_4_–shell (less intensity) indicated for arrows. (d) Pb and O elemental distribution across the nanoparticle.

The chemical composition was analyzed by EDS. Figure 4a shows the linear scanning EDS analysis of the nanoparticles in the square. In Figure 4b, the EDS spectrum profile shows the presence of lead and oxygen in the nanoparticle. Furthermore, it is possible to trace the chemical composition along the core–shell nanoparticle from EDS. Figure 4c shows the intensity profile of the EDS spectrum, where the different signal intensities can be seen; in the center of the nanoparticle, the number of counts obtained was higher due to the Pb core, while at the edges, the number of counts was lower due to the presence of oxygen in this part of the nanoparticles (arrows indicate this area). Figure 4d shows the elemental distribution of Pb and O across the nanoparticle. The high intensity of Pb (metallic) counts in the center of the nanoparticles is observed.

**Figure 5:**
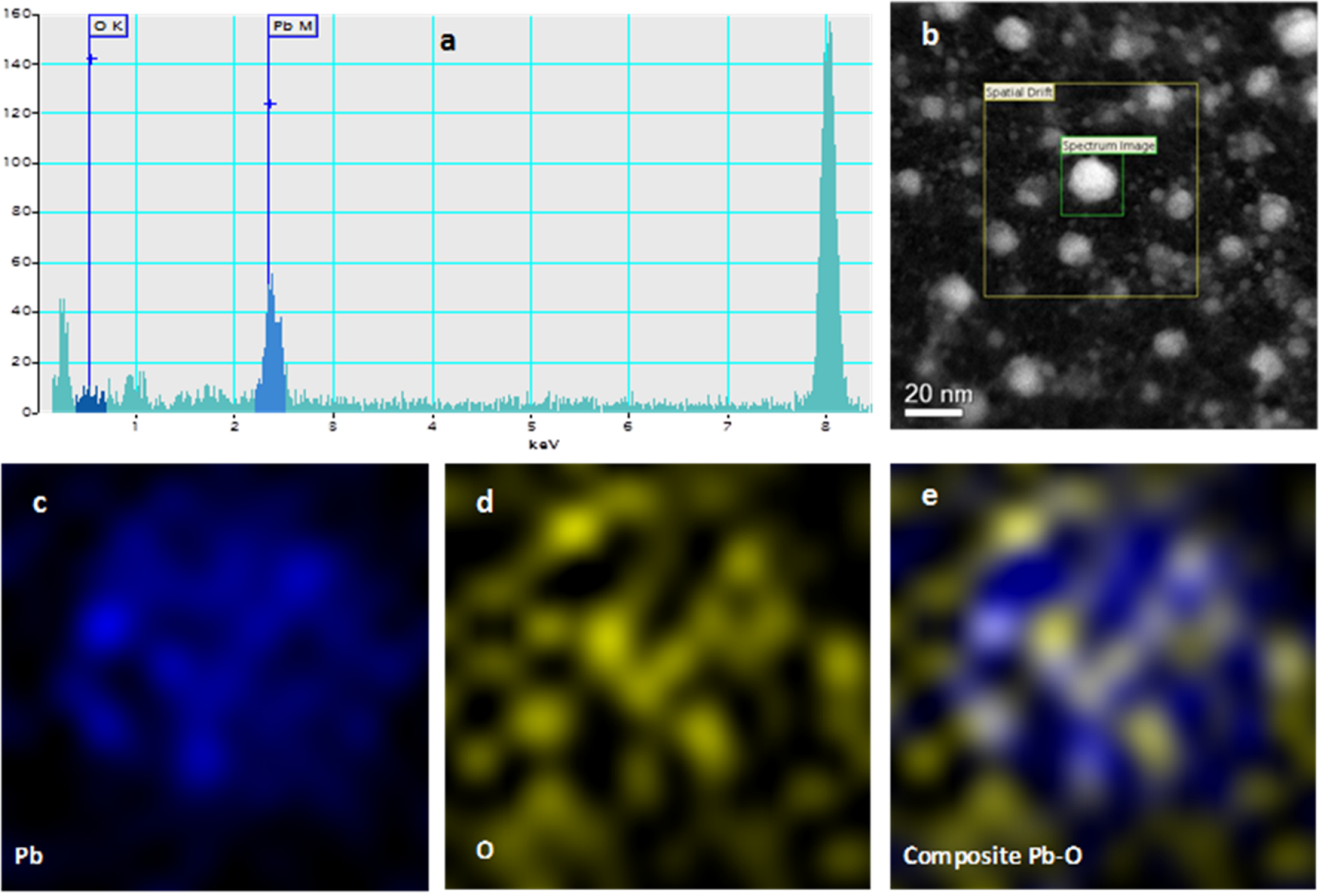
(a, b) Element mapping by EDS. (c–e) EDS mapping for Pb, O, and the Pb–O compound, respectively.

Figure 5 shows the EDS mapping analysis of the nanoparticle. The EDS spectrum (5a) shows the characteristic energy lines of Pb and O from the nanoparticle image marked in the inset (5b). Figure 5c,d shows the elemental mapping analysis for Pb and O, respectively. This analysis shows that lead atoms are mainly in the center of the nanoparticle, while oxygen atoms are all over the nanoparticle. It is a two-dimensional (2D) view, but in a three-dimensional (3D) perspective, lead atoms would occupy the center, and oxygen would be on the nanoparticle’s surface, corroborating previous analyses. (e) elemental mapping analysis of the Pb/Pb_3_O_4_ nanocomposite.

**Figure 6:**
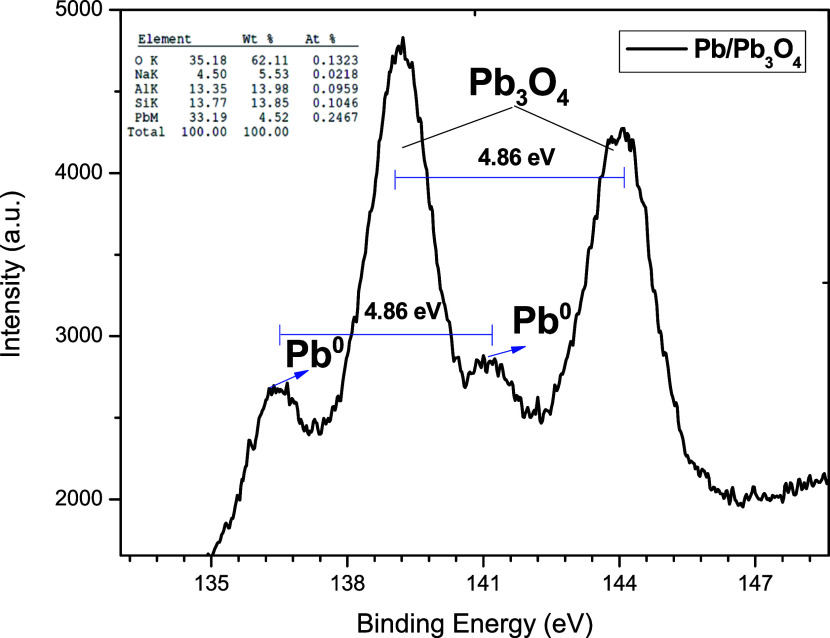
XPS spectra of the Pb and Pb_3_O_4_ contribution of the Pb/Pb_3_O_4_ system.

Analysis of the XPS spectrum in Figure 6 showed the presence of lead and lead oxide. The Pb ion maintains the divalent state with a binding energy of 4f_7/2_ and 4f_5/2_ of 138.8 and 143.6 eV associated with oxidized lead, respectively.^35,36^ In addition, the binding energies 136.4 and 141.2 eV are associated with Pb^0^ 4f_7/2_ and Pb^0^ 4f_5/2_, respectively.^37^ Zhao et al. reported an XPS spectrum of Pb 4f composed of two peaks corresponding to metallic Pb at 137.0 eV and Pb_3_O_4_ at 138.4 eV.^38^ Likewise, a difference of 4.8 eV of the binding energy differences between Pb 4f_7/2_ and Pb 4f_5/2_ of Pb^0^, Pb–O was obtained. The results are similar to those reported in the literature.^36,39,40^ From the XPS results, Pb^0^ and Pb–O coexist in the same structure. In addition, it is observed that the lead oxide content is higher than that of Pb^0^ due to the core–shell configuration. The results are consistent with the analyses of the obtained TEM and SEM images obtained.

**Figure 7:**
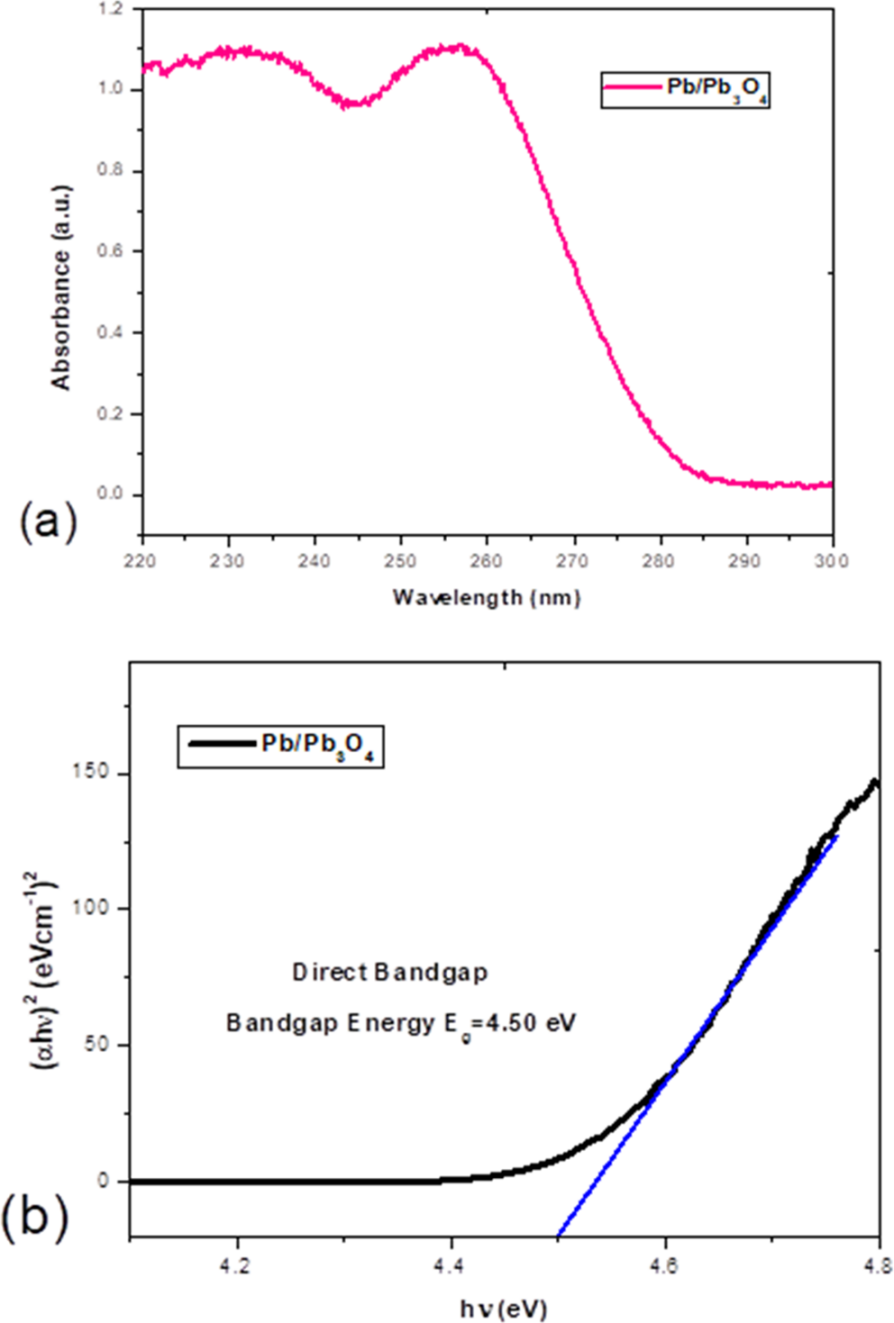
(a) UV–Vis absorption spectra of Pb/Pb_3_O_4_ (b) Calculation of the optical band gap from UV–vis absorption spectra.

The UV–vis spectrum obtained shows a broad band with two important contributions (Figure 7). The energy bands of the semiconductor and the properties of the energy states of the metal influence the absorption spectrum of a metal/semiconductor alloy. Depending on the doping levels and the crystal structure of the alloy, additional absorption peaks may appear in the spectrum. These peaks may be associated with specific electronic transitions in the semiconductor or with surface plasmons in the metal.^41−43^ In Figure 7a, the first band centered at 235 nm is attributed to the Pb nanoparticles’ surface plasmon resonance (SPR). This result is close to a previous report for the clinoptilolite-Pb.^30^ The spectrum indicates that Pb^0^ has a high visible light absorption capacity, which significantly impacts the material’s photophysical and photochemical properties. On the other hand, a fundamental contribution is observed in the absorption band centered at 256 nm. This band is attributed to lead oxide nanoparticles.^44,45^Figure 7b shows the band gap value obtained for the shell, with a value of 4.50 eV. The band gap value for the semiconductor contribution was calculated by plotting the square of (α*h*ν) against *h*ν and extrapolating the linear part of the curve until reaching (α*h*ν)^2^ = 0. Here, α represents the absorption coefficient and *h*ν represents the photoenergy.^46^ Lead oxide (Pb_3_O_4_) has a band gap in the range of 1.9 to 2.1 eV, which classifies it as a semiconductor material with a comparatively narrow energy gap (eV).^47^ Combining lead (Pb) and lead oxide (Pb_3_O_4_) in the nanocomposite structure could result in a wider band gap. Other literature reports have observed this effect by doping Pb or PbO with other compounds.^48−50^ In addition, the core–shell configuration of the nanocomposite implies that the particle is not a pure semiconductor. The Pb core and the small width of the shell can affect the electronic properties. Smaller particles may have a higher band gap due to quantum confinement effects.^51^ Our result is consistent with the concept that the band gap increases as the particle size decreases. Due to the proximity of the electron–hole pairs, the Coulombic interaction between them becomes significant and cannot be ignored, increasing the kinetic energy.^52,53^

## Conclusions

The Pb/Pb3O4 nanocrystalline structures were obtained by a relatively simple procedure using zeolite 4A. The composite nanostructure was analyzed in detail using HRTEM, BF, HAADF-STEM, and XPS techniques. The Pb/Pb_3_O_4_ nanoparticles showed a well-defined core–shell structural configuration with 5 and 25 nm diameters. HRTEM analysis showed that the core corresponds to the Pb FCC crystal. Meanwhile, the atomic arrangement of the shell corresponds to a tetragonal Pb_3_O_4_ crystal.

Additionally, HAADF-STEM shows the different components of the core and surface of nanoparticles. EDS analysis confirms the composition difference between the particles’ interior and surface, indicating the presence of Pb (core) and Pb–O (shell). XPS and UV–vis spectra showed lead and lead oxide coexistence in the same structure. The band gap obtained for the shell was 4.50 eV. Finally, it is important to apply low-cost synthesis methods to obtain new metal–semiconductor nanocrystals. Lead and lead oxide nanoalloys can be used in optoelectronic devices such as solar cells, photodetectors, and light-emitting diodes (LEDs). It is the first time a Pb/Pb_3_O_4_ nanocomposite has been obtained, which opens possibilities to apply this synthesis method to obtain other composite nanomaterials.
